# Artemvulactone E isolated from *Artemisia vulgaris* L. ameliorates lipopolysaccharide-induced inflammation in both RAW264.7 and zebrafish model

**DOI:** 10.3389/fphar.2024.1415352

**Published:** 2024-07-18

**Authors:** Zibo Zhao, Shimin Lin, Tao Liu, Xiao Hu, Shurong Qin, Fengyun Zhan, Jiaqi Ma, Chen Huang, Zhibin Huang, Yifei Wang, Kai Zheng, Wenqing Zhang, Zhe Ren

**Affiliations:** ^1^ Department of Cell Biology, College of Life Science and Technology, Jinan University, Guangzhou, China; ^2^ Guangdong Province Key Laboratory of Bioengineering Medicine, Jinan University, Guangzhou, China; ^3^ Guangdong Provincial Biotechnology Drug and Engineering Technology Research Center, Jinan University, Guangzhou, China; ^4^ National Engineering Research Center of Genetic Medicine, Jinan University, Guangzhou, China; ^5^ Key Laboratory of Innovative Technology Research on Natural Products and Cosmetics Raw Materials, Jinan University, Guangzhou, China; ^6^ Division of Cell, Developmental and Integrative Biology, School of Medicine, South China University of Technology, Guangzhou, China; ^7^ National Engineering Technology Research Center for Modernization of Traditional Chinese Medicine, Jinan University, Guangzhou, China; ^8^ School of Pharmacy, Shenzhen University Medical School, Shenzhen University, Shenzhen, China

**Keywords:** inflammation, artemvulactone E, TLR4, zebrafish, molecular dynamics

## Abstract

**Introduction:**

Natural plants are valuable resources for exploring new bioactive compounds. *Artemisia vulgaris* L. is a traditional Chinese medicinal herb that has been historically used for treating multiple diseases. Active compounds isolated and extracted from *A. vulgaris* L. typically possess immunomodulatory and anti-inflammatory properties. Artemvulactone E (AE) is a new sesquiterpene lactone isolated and extracted from *A. vulgaris* L. with unclear biological activities.

**Methods:**

The immunoregulatory effects of AE on macrophages were assessed by ELISA, RT-qPCR, immunofluorescence, and western blot assay. The effect of AE on lipopolysaccharide (LPS) -relates signaling pathways was examined by western blot assay. In zebrafish models, the larvae were yolk-microinjected with LPS to establish inflammation model and the effect of AE was evaluated by determining the survival rate, heart rate, yolk sac edema size, neutrophils and macrophages infiltration of zebrafish. The interaction between AE and Toll-like receptor 4 (TLR4) was examined by molecular docking and dynamic stimulation.

**Results:**

AE reduced the expression and secretion of pro-inflammatory cytokines (TNF-α and IL-6), inflammatory mediators iNOS and COX-2, as well as decreases the production of intracellular NO and ROS in LPS-stimulated macrophages. In addition, AE exerted its anti-inflammatory effect synergistically by inhibiting MAPK/JAK/STAT3-NF-κB signaling pathways. Furthermore, AE enhanced the survival rate and attenuated inflammatory response in zebrafish embryos treated with LPS. Finally, the molecular dynamics results indicate that AE forms stable complexes with LPS receptor TLR4 through the Ser127 residue, thus completely impairing the subsequent activation of MAPK-NF-κB signaling.

**Conclusion:**

AE exhibits notable anti-inflammatory activity and represents as a potential agent for treating inflammation-associated diseases.

## Introduction

Inflammation is a self-limiting biological process that is triggered by immune system to prevent abnormal damages and aimed at recognizing and diminishing pathogens, as well as maintaining normal tissue homeostasis (Panigrahy et al., 2021; Zarrin et al., 2021). During the immune defense process, a plethora of inflammatory cytokines is produced in the body and the acute inflammatory response is initiated by various pathogens or trauma, which may occur in a matter of minutes to hours and persist for days or even weeks (Castanheira and Kubes, 2019). Nonetheless, an immoderate inflammatory reaction is known as the primary reason for acute and chronic inflammation, encompassing inflammatory bowel disease, vascular diseases, cancer and rheumatoid arthritis (Libby, 2021; Sanmarco et al., 2022). Chronic inflammation is prevalent, long-lasting and affects numerous patients, resulting in a considerable economic burden on society and individuals (Leuti et al., 2020). Clinical treatment of inflammation often leads to severe cardiovascular and gastrointestinal side effects, highlighting the need for the development of novel, safe and effective anti-inflammatory medications.

Due to the less toxic side effects and potent biological activity offered by natural medicines, screening and developing fresh anti-inflammatory drugs is a crucial direction of the ongoing research and development into anti-inflammatory drugs (Soehnlein and Libby, 2021). *A. vulgaris* L. is a traditional Chinese medicine that is extensively applied to address inflammatory illnesses inclusive of hepatitis, enteritis, and gastric ulcer (Rayego-Mateos and Valdivielso, 2020; Soehnlein and Libby, 2021; Kronsten et al., 2022). The complex chemical composition of *A. vulgaris* L. has resulted in a diverse range of biological activities, encompassing anti-inflammatory, immunomodulatory, anti-tumour, antimutagenic, antibacterial, and anticoagulant effects (Dib and El Alaoui-Faris, 2019; Ekiert et al., 2020; Singh et al., 2023). Certain extracts and volatile oil derived from *A. vulgaris* L. possess anti-inflammatory properties. However, due to the complex nature of their components, low levels of active compounds and unclear mechanisms of action, research investigating the anti-inflammatory activity of *A. vulgaris* L. has shifted towards highlighting the effects of monomers. In our previous research, we isolated a new sesquiterpene lactone, Artemvulactone E (AE), from *A. vulgaris* L. Our initial findings suggested the AE to possess anti-inflammatory properties (Liu et al., 2022). Nonetheless, the *in vivo* anti-inflammatory efficacy of AE requires evaluation.

LPS can induce excessive production of various proinflammatory cytokines, leading to organ and tissue damage (Elenkov and Chrousos, 1999; Donath and Shoelson, 2011). Both the mitogen-activated protein kinase (MAPK) pathways, consisted of the ERK, JNK and p38 MAPK family members, and the Janus kinase (JAK)-signal transducer and activator of transcription (STAT) pathway are important mediators of LPS-stimulated inflammatory response (Heinrich et al., 2003; Kamimura et al., 2003). LPS stimulates the MAPKs and JAK/STAT signaling pathway to activate NF-κB, an essential transcription factor for the transcription of many proinflammatory genes (Aiba and Nakamura, 2013; Liu et al., 2017). This leads to the fact that LPS stimulation can over-activate cyclooxygenase-2 (COX-2), promote the production of prostaglandins (e.g., PTGS2), and activate inducible nitric oxide synthase (iNOS, encoded by the NOS2 gene) to produce NO. In conclusion LPS activates natural immunity by binding to TLR4 to activate the Myd88-TRAF6-TAK1 complex, which in turn triggers the MAPK and NF-κB signaling pathways (He et al., 2016). This leads to the fact that LPS stimulation can over-activate cyclooxygenase-2 (COX-2), promote the production of prostaglandins (e.g., PTGS2), and activate inducible nitric oxide synthase (iNOS, encoded by the NOS2 gene) to produce NO. In conclusion LPS activates natural immunity by binding to TLR4 to activate the Myd88-TRAF6-TAK1 complex, which in turn triggers the MAPK and NF-κB signaling pathways (He et al., 2016).

In the present study, we initially investigated the anti-inflammatory impact of AE on RAW 264.7 cells and subsequently studied its effects in zebrafish. Subsequently, we concentrated on analyzing the influence of AE on proteins that are significant for inflammation and elucidating the mechanisms by which AE boosts its anti-inflammatory impacts.

## Materials and methods

### Plant material

Fresh leaves of *A. vulgaris* L. (Asteraceae) were harvested in May 2018 in Tangyin (Henan Province, China; GPS coordinates: E 11425′41.05; N 3552′7.38) and air dried at room temperature in the shade. Professor Yifei Wang (College of Life Science and Technology, Jinan University, Guangzhou, China) identified the plant samples (No. 201805). Specimen vouchers were deposited at Jinan Biomedicine Research and Development Centre, Jinan University (Guangzhou, China).

### Cells and reagents

Dulbecco’s Modified Eagle Medium (DMEM, C11995500BT, Gibco), fetal bovine serum (FBS, A5669801) and proteinase K (A5669801) were purchaseed from Thermo Fisher Scientific (Waltham, United States). Raw264.7 cells, purchased from the American type culture collection (TIB-71, ATCC, Manassas, VA, United States), were cultured in DMEM containing 10% FBS and at 37°C in 5% CO2 incubator.

Lipopolysaccharide (LPS, L2880) (*E. coli*, O55: B5) and BCIP/NBT were gained from Sigma-Aldrich (St. Louis, United States) and dexamethasone (DEX) was from Jinyao (HY-14648, Tianjin, China). TNF-α (SZB-EK0525) and IL-6β (SZB-EK0308) enzyme-linked immunosorbent assay (ELISA) kits were purchased from Sizhengbai (Beijing, China). Nuclear and cytoplasmic protein extraction kit (P0027), enhanced chemiluminescence (ECL, P0018), reactive oxygen species assay kit (S0033), nitric oxide assay kit (S0021) and DAF-FM DA probe (S0019S) were gained from Beyotime (Shanghai, China). Antibody against iNOS (13120), COX-2 (12282), p-ERK1/2 (9101), ERK1/2 (4695), IKK-β (8943), p-IKKα/β (2697), IκBα (4812), p-IκBα (2859), p-p65 (3033), p65 (8242), Histone 3, p-JAK2 (3771), JAK2 (3230), p-JNK (9251), JNK (9252), p-STAT3 (9145) and STAT3 (9139) were puchased from Cell Signaling Technology (Danvers, United States) and GAPDH antibody (GTX100118) was obtained from Genetex (California, United States). Alexa Fluor 488-conjugated antiRabbit IgG antibody (A-11008) was obtained from Invitrogen (Grand Island, United States). MAB block reagent (11096176001) and Anti-Digoxigenin-AP (11093274910) were purchased from Roche (Basel, Switzerland).

### Cell viability assay

The cytotoxicity of AE on Raw264.7 cells were measured using the MTT assay (475989, Sigma, United States). The cells were plated in 96-well plates with 2.0 × 10^4^/well and were incubated with different concentrations (1.563, 3.125, 6.25, 12.5, 25, 50, 100, and 200 μM) of AE for 12 h and 24 h, followed by the MTT procedures, respectively. A multimode reader (Bio-Rad, Hercules, CA, United States) was used to record the 570 nm absorbance. The 50% cytotoxic concentration (CC_50_) was also calculated.

### Quantitative real time PCR

Raw264.7 cells were exposed to LPS (200 ng/mL) and different concentrations of AE simultaneously for 12 h. RNA extracted from Raw264.7 cells was used for RNA-to-cDNA transcription in the quantitative PCR assay. RT-qPCR analysis was performed with SYBR Green Premix Pro Taq HS qPCR kit (172-5260, Bio-Rad, Hercules, CA, United States) on a CFX96 Touch Real-Time PCR Detection System (1855201, Bio-Rad, Shanghai, China). The fold changes of target genes was normalized to the expression level of GAPDH. The primer sequences are shown in Supplemental Table S1.

### Western blot

RIPA buffer (C1005, Beyotime, Shanghai, China) was used to extract proteins in Raw264.7 cells and a BCA protein assay kit (P0012, Beyotime) was used to measure the concentration. The protein samples were separated by SDS-PAGE and transferred onto a PVDF membrane. Afterwards, a buffer consisting of 5% BSA was utilized to block the membrane for 1 h, followed by an incubation of the membrane with primary antibodies at 4°C overnight. HRP-coupled secondary antibody was subsequently incubated with the protein membrane for 1 h, the results of which were subjected to further analysis by a ChemiDoc MPTM Imaging System (12003154, Bio-Rad, Hercules, CA, United States).

### ELISA assay

Raw264.7 cells were exposed to LPS (200 ng/mL) and different concentrations of AE simultaneously for 24 h. Then levels of IL-6 and TNF-α in cell supernatants were measured by the specific ELISA kit, following the manufacturer’s instructions.

### Extra-cellular no content assay

Griess reagent was used to evaluate no levels. Supernatant of different groups were sequentially treated with equal volume of Griess reagent I and reagent II respectively, and the 540 nm absorbance was recorded by a multimode reader (1681130, Bio-Rad, Hercules, CA, United States).

### Determination of intracellular ROS

After AE and LPS treatment, Raw264.7 cells were incubated with 2 mL of probe working solution (DCFH-DA: DMEM = 1:1,000) in the dark at 37°C for 20 min. The cells were then washed three times with PBS and were fluorescently photographed using the FITC parameter setting in the inverted fluorescence microscope (GTX100118, Nikon, Tokyo, Japan).

### Immunofluorescence

Raw264.7 cells were exposed to LPS (200 ng/mL) and AE for 12 h, and then 4% paraformaldehyde (PFA, P6148, Sigma-Aldrich, United States) was added and fixed for 20 min, followed by permeabilization for 15 min. After blocking with 5% BSA for 1 h, p65 antibody (p65:PBST = 1:300) was added and incubated at 4°C overnight. Alexa Fluor 488-conjugated antiRabbit IgG antibody (antibody:PBST = 1:1,000) was added and incubated for 1 h in the dark, and DAPI solution (62248, Thermo Fisher Scientific, United States) was added for staining for 15 min. Finally, fluorescence confocal microscope LSM700 (Zeiss, Oberkochen, German) was used to perform confocal microscopy assay.

### Zebrafish maintenance

AB strain zebrafish was kindly provided by the medical school of South China University of Technology and raised at 28°C with a 14:10 h light: dark cycle in a recirculating tank system using local tap water (pH 7.2–7.6, salinity 0.03%–0.04%). Before the experiment, zebrafish were randomly selected for interbreeding in a male-to-female ratio of 1:2. The embryos were collected and maintained in egg water containing 5% methylene blue as a fungicide. To prevent pigmentation, embryos were incubated in egg water containing 0.003% 1-phenyl-2-thiourea (PTU, P7629, Sigma-Aldrich, United States) at 24 hpf.

### Toxicity evaluation of AE on zebrafish

Zebrafish embryos at 3 days post fertilization (dpf) were randomly divided into a 12-well plate (n = 20) and then immersed in different concentrations of AE (1.563–25.00 μg/mL). Culture medium was changed daily. Dead embryos were recorded and removed at every 24 h intervals until the 6th dpf.

### LPS microinjection inflammatory model and drug administration

The zebrafish larvae at 3 dpf were anesthetized with 0.02% tricaine and immobilized in a clear Petri dish coated with 1.5% agarose (A9539, Sigma-Aldrich, United States). Subsequently, the larvae were yolk-microinjected with 2 nL of LPS (0.5 mg/mL) to establish inflammation model by Cell Microinjector (Micro Data Instrument, Plainfield, United States). PBS was used as the negative control. After recovering from anesthesia, these larvae were randomly divided into a 12-well plate with AE (1, 2, 4 μM) or DEX (5 μM) in 3 mL egg water, and incubated at 28.5°C for particular periods. The survival rate of zebrafish larvae in each group was recorded every 12 h until 72 h post injection (hpi).

### Measurement of heart beating rate and yolk sac edema size

Larvae were anesthetized at 9 hpi and plated on a clear Petri dish coated with 1.5% agarose, then count and record the number of atrial and ventricular beats for 3 min under the microscope (Zeiss, Oberkochen, German), and the results are expressed as the average heart rate per minute. For the size measurement of yolk sac edema at 9 hpi after LPS-exposure, lateral views of anesthetized embryos were imaged using a stereomicroscope (Zeiss, Oberkochen, German). The contour of the yolk sac edema was traced, and the area within each tracking range was determined by MoticImages Plus 3.0 (Motix, Xiamen, China).

### Histopathological observation of zebrafish larvae

Three dpf larvae were treated with AE or DEX for 9 h after LPS microinjection. Then, they were fixed with 4% PFA, dehydrated with graded ethanol (108,543, Sigma-Aldrich, United States) and embedded with paraffin (100496, Sigma-Aldrich, United States). After that, larvae specimens were cut into 4 μm thin sections, deparaffinized and stained with hematoxylin and eosin (H&E, 818715, Sigma-Aldrich, United States), each tissue section was observed under a light microscope (Olympus, Tokyo, Japan).

### Sudan black staining

3 dpf larvae were treated with AE or DEX for 9 h after LPS microinjection. The larvae were then fixed with 4% paraformaldehyde for 2 h at room temperature, washed three times with PBST, and then stained with Sudan black (SB) solution (199664, Sigma-Aldrich, United States) for 30 min. After that, the larvae were extensively washed with 70% ethanol, and then guadually rehydrated with PBST twice. SB positive cells were observed and counted under a stereomicroscope (Zeiss, Oberkochen, German).

### Whole-mount *in situ* hybridizations in zebrafish embryos

Three dpf larvae were treated with AE or DEX for 9 h after LPS microinjection. Embryo pretreatment: The larvae were fixed with 4% PFA for 2 h at room temperature, rinsed twice with PBST, dehydrated successively with 50% methanol solution and 100% methanol solution, and placed in fresh 100% methanol solution at −20°C overnight. Rehydration and hybridization: the larvae were dehydrated successively with 75%, 50%, and 25% methanol, rinsed with PBST, digested with proteinase K for 15–20 min, fixed with 4% PFA for 20 min, rinsed with PBST twice, prehybridization with HB balance for 5 min at 64°C, and hybridized with probes (1.5 μg/mL) at 64°C overnight. Incubation of antibodies: the larvae were washed twice with SSCT solution (S6639, Sigma-Aldrich, United States) containing 50% formamide, 2 × SSCT solution, 0.2 × SSCT solution and PBST, incubated with blocking buffer (2% LS in PBST) at room temperature for 1 h, and then incubated with Anti-Digoxigenin-AP (1:2,000 in blocking buffer) at room temperature for 2 h. Coloration: the larvae were washed with PBST three times, incubated with Buffer 9.5T twice, colored with NBT/BCIP dye until obvious signal is observed under the microscope, rinsed with PBST twice, fixed with 4% PFA for 1 h at room temperature, and the larvae were stored in 70% glycerol at 4°C. Mfap4 positive cells were observed and counted under a stereomicroscope (Zeiss, Oberkochen, German).

### Molecular docking and binding energy

Firstly, the 3D structure of the AE substance was constructed in Chemdraw 3D and energy minimization is performed under the MM2 force field to obtain the input file of the ligand. The 3D conformation file of the proteins was obtained from the RCSB-PDB database. Imported the ligand and receptors into Schrödinger, and searched for ligand conformations under the OPLS4 force field using the Ligprep. Then protein dehydration, hydrogenation, optimization and energy minimization used the protein preparation function block. Binding pockets were determined using Sitemap. Finally, molecular docking was carried out, and the binding energy was calculated.

### Molecular dynamics simulation

First, the protein is pre-processed, using the OPLS4 force field representation, and converted into an input file for use with Desmond. The TIP3P water model was used to perform the entire simulation, which lasted 100 ns. The pressure and temperature were maintained at a constant 1 bar respectively during the equilibrium simulation of the MD simulation, and finally the results were checked and displayed using the Python tool.

### Statistical analysis

Statistical analyses were performed using GraphPad Prism. Statistical comparisons were made using the two-tailed Student’s t-test for two experimental groups or the one-way analysis ofvariance (ANOVA) for multiple groups. Dunnett’s test was used as the *post hoc* analysis following ANOVA. The control group’s mean value was compared to that of the LPS group, with #*p* < 0.05, ##*p* < 0.01, ###*p* < 0.001, and ####*p* < 0.0001 indicating significant distinctions, while ns indicated no significant difference. The mean value of the administration group was compared to that of the LPS group, where *p < 0.05, **p < 0.01, ***p < 0.001, and ****p < 0.0001 showed significant differences, and ns represented no significant difference. All experimental data were expressed as mean ± SEM. A t-test was used for the statistical analysis (n = 3).

## Results

### Cytotoxicity analysis of AE

**FIGURE 1:**
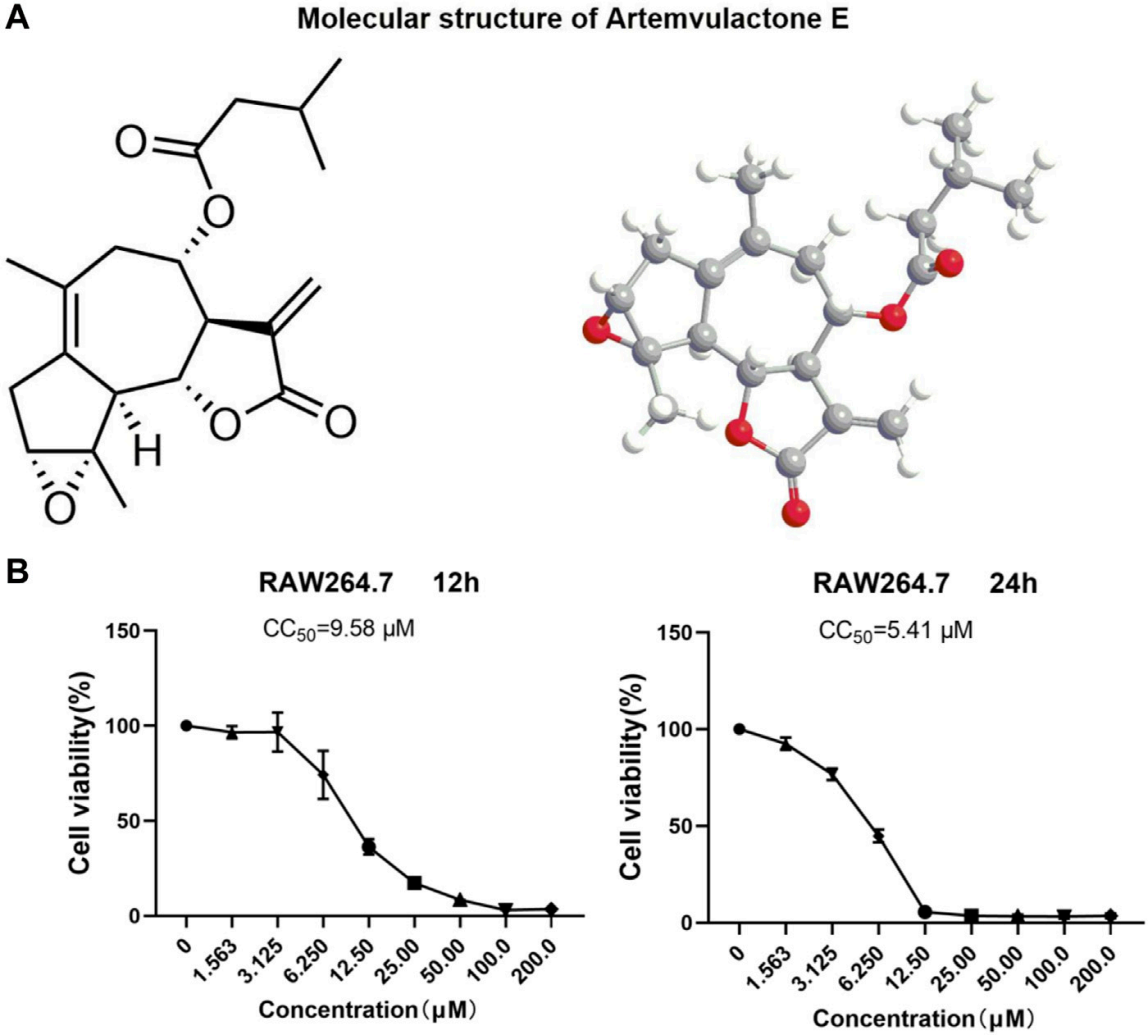
Structure and cytotoxicity of AE. **(A)** Molecular structure of AE. **(B)** RAW264.7 cells were treated with AE at different concentrations for 12 h and 24 h and cell viability was determined.

The sesquiterpene lactone AE extracted from *A. vulgaris* L. was guaiacol (Figure 1A), and was more than 98% pure (Supplementary Figure S1). An MTT assay was conducted to determine the non-toxic concentration of AE on RAW264.7 cells. The results showed a concentration-dependent toxicity of AE on RAW264.7 cells with CC_50_ values of 9.58 μM for 12 h and 5.41 μM for 24 h (Figure 1B). Therefore, in subsequent experiments, a concentration gradient of 0.5, 1, and 2 μM was used as the working concentration. These concentrations were selected based on preliminary experiments, covering a range from sub-cytotoxic to potentially therapeutic levels. The purpose of choosing these concentrations was to observe the dose-dependent effects of AE on cell viability. Lower and intermediate concentrations (0.5 and 1 μM) are expected to exhibit minimal cytotoxicity, serving as control groups to evaluate the baseline effects of AE, while a higher concentration (2 μM) is anticipated to show more pronounced effects, allowing us to assess the potential cytotoxicity and therapeutic threshold of AE.

### AE exhibits anti-inflammatory effects in LPS-stimulated RAW264.7 cells

**FIGURE 2:**
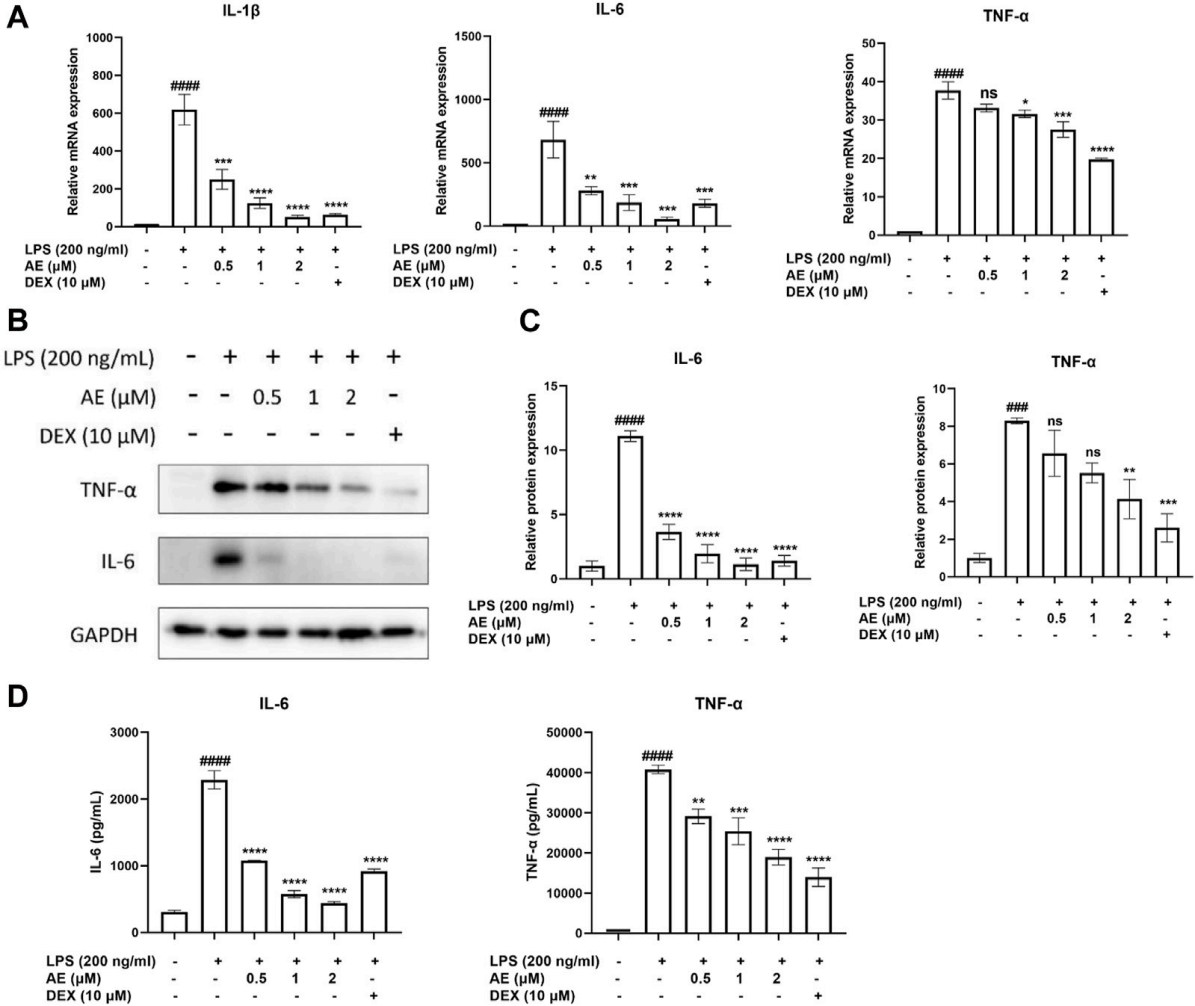
The anti-inflammatory effects of AE on RAW 264.7 cells. **(A)** RAW264.7 were treated with LPS and different concentration of AE for 12 h and then RT-qPCR was performed. **(B)** RAW264.7 were treated with LPS and AE for 12 h, and then Western blot was performed. **(C)** Relative protein expression levels of TNF-α and IL-6. **(D)** RAW264.7 were treated with LPS and AE for 24 h, and TNF-α and IL-6 release was measured in the culture supernatants. Data were represented as mean ± SEM (n = 3), ^*^ *p* < 0.05; ^**^ *p* < 0.01; ^***^ *p* < 0.001;^****^ *p* < 0.0001; ns, no significance, compared with each’group. ^###^ *p* < 0.001; ^####^ *p* < 0.0001; compared with Ctrl group.

Next, we examined the effect of AE on the mRNA expression of proinflammatory gene TNF-α, IL-1β, IL-6. As shown in Figure 2A, LPS stimulation significantly increased the mRNA expression level of proinflammatory genes in LPS-stimulated RAW264.7 cells, which were remarkably inhibited by AE. In addition, AE effectively inhibited the protein expression and release of TNF-α [*p* < 0.01, F (5, 12) = 12.16] and IL-6 [*p* < 0.0001, F (5, 12) = 57.60] in concentration-dependent manner (Figures 2B–D), indicating that AE had certain anti-inflammatory activity *in vitro*. The results are superior or close to DEX.

### AE suppresses the expression of iNOS and COX-2

**FIGURE 3:**
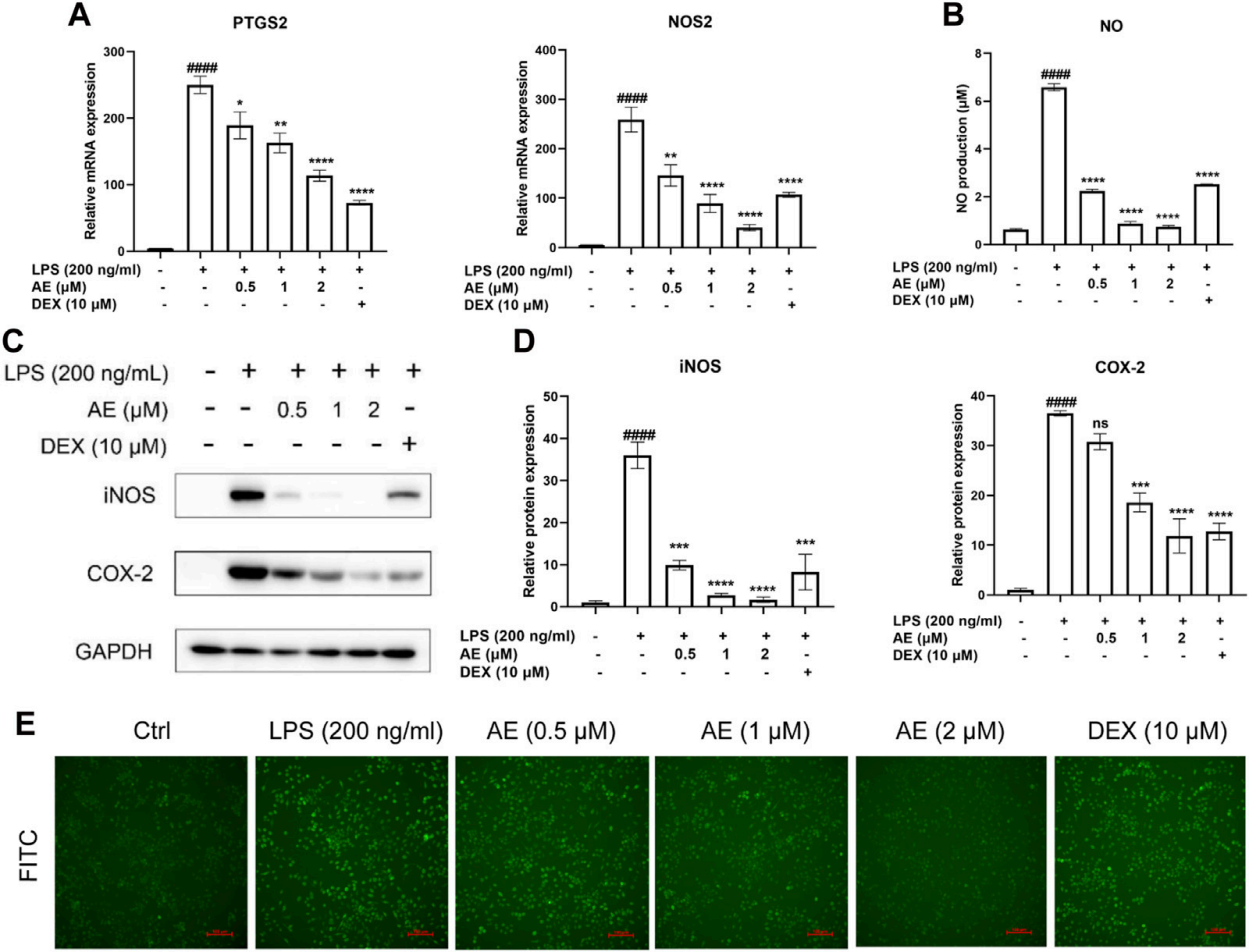
AE decreases LPS-stimulated iNOS, COX-2 and ROS generation. **(A)** Changes in mRNA level expression of NOS2 and PTGS2. **(B)** NO release was measured in the culture supernatants by Griess reagent. **(C)** RAW264.7 were treated with LPS and AE for 12 h, and then Western blot was performed. **(D)** Relative protein expression levels of iNOS and COX-2. **(E)** The ROS generation level RAW264.7 were treated with LPS and AE for 12 h. Data were represented as mean ± SEM (n = 3), ^*^ *p* < 0.05; ^**^ *p* < 0.01; ^***^ *p* < 0.001;^****^ *p* < 0.0001; ns, no significance, compared with each’group. ^##^ *p* < 0.01; ^###^ *p* < 0.001; ^####^ *p* < 0.0001; compared with Ctrl group.

We found that 2 µM AE significantly reduced the mRNA expression levels of NOS2 [*p* < 0.0001, F (5, 12) = 32.82] and PTGS2 [*p* < 0.0001, F (5, 12) = 53.41] (Figure 3A), and the release of NO (*p* < 0.0001) (Figure 3B). Consistently, AE also markedly downregulated the protein levels of COX-2 [*p* < 0.0001, F (5, 12) = 48.53] and iNOS [*p* < 0.0001, F (5, 12) = 35.36] (Figures 3C, D), and its inhibitory effect was superior to that of DEX. Considering that LPS can stimulate reactive oxygen species (ROS) production through the NAPDH oxidase pathway, leading to severe oxidative damage and inflammatory cascade reaction, We assessed ROS production using DCFH-DA staining and observed that AE treatment more effectively reduced DCFH-DA immunofluorescence intensity compared to the DEX group, indicating a stronger inhibition of ROS production (Figure 3E). Therefore, these results indicate that AE ameliorates inflammation and oxidative damage caused by LPS.

### MAPKs and JAK2/STAT3 pathways are involved in AE-mediated protection

**FIGURE 4:**
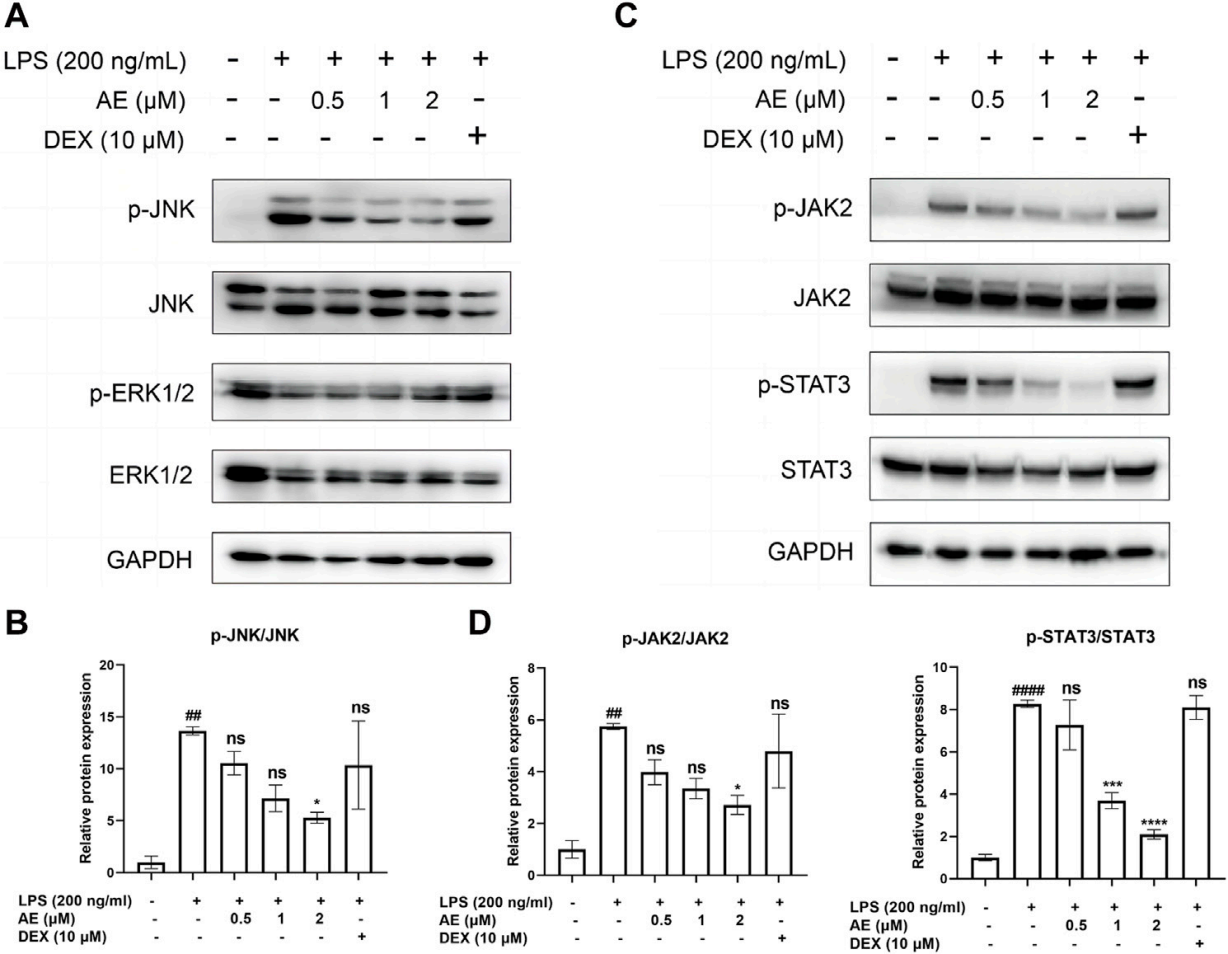
Inhibitory effects of AE on the activation of MAPK and STAT3. **(A,C)** RAW264.7 were treated with LPS and AE for 12h, and Western blot was then performed **(B,D)** Relative protein expression levels of p-JNK, or p-JAK2 and p-STAT3. Data were represented as mean ± SEM (n = 3), ^*^ *p* < 0.05; ^**^ *p* < 0.01; ^***^ *p* < 0.001;^****^ *p* < 0.0001; ns, no significance, compared with each’group. ^##^ *p* < 0.01; ^###^ *p* < 0.001; ^####^ *p* < 0.0001; compared with Ctrl group.

We found that LPS stimulation upregulated the phosphorylation level of JNK [*p* < 0.01, F (5, 12) = 5.596], whereas 2 µM AE treatment significantly inhibited (*p* < 0.05) this effect compared to DEX (*p* > 0.05) treatment (Figures 4A, B). Additionally, LPS markedly induced the phosphorylation of JAK2 and STAT3, while AE treatment further suppressed JAK2 [*p* < 0.05, F (5, 12) = 6.140] and STAT3 [*p* < 0.0001, F (5, 12) = 31.72] phosphorylation more effectively than DEX (*p* > 0.05) treatment (Figures 4C, D). These results indicated that AE inhibits the MAPK and JAK/STAT3 pathway to exert its anti-inflammatory effect.

### AE inhibits the activation of nuclear factor-κB (NF-κB) signaling

**FIGURE 5:**
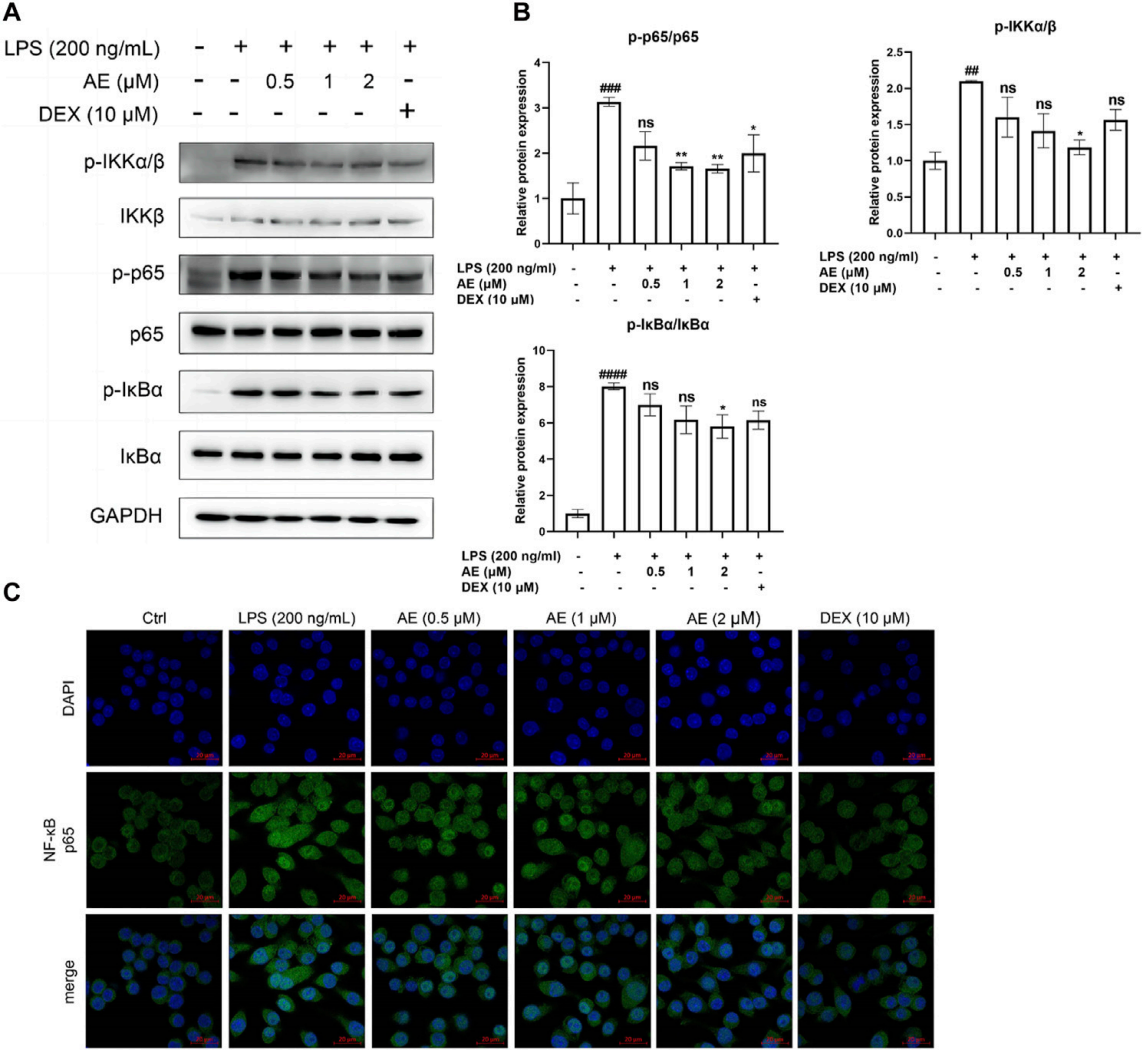
AE inhibits the activation of NF-κB. **(A)** RAW264.7 were treated with LPS and AE for 12 h, and Western blot was then performed. **(B)** Relative protein expression levels of p-IKKα/β, p-p65 and p-IκBα. **(C)** The cells were treated with LPS and AEfor 12 h, NF-κB p65 was immunostained with anti-p65 antibody and detected using secondary antibody conjugated with Alexa FluorTM 488 (green) followed by DAPI (blue) staining for nuclei. Scale bar, 20 μm. Data were represented as mean ± SEM (n = 3), ^*^ *p* < 0.05; ^**^ *p* < 0.01; ^***^ *p* < 0.001;^****^ *p* < 0.0001; ns, no significance, compared with each’group. ^##^ *p* < 0.01; ^###^ *p* < 0.001; ^####^ *p* < 0.0001; compared with Ctrl group.

As shown in Figures 5A, B, LPS stimulation significantly increased the phosphorylation level of key proteins IKK [*p* < 0.01, F (5, 12) = 4.919], IκBα [*p* < 0.0001, F (5, 12) = 20.79] and NF-κB p65 [*p* < 0.001, F (5, 12) = 7.325] of NF-κB signaling pathway, whereas AE inhibited such activation (*p* < 0.05, *p* < 0.05, *p* < 0.001). In addition, by performing immunofluorescent assay, we found that most of NF-κB p65 was translocated to the nucleus in LPS-stimulated RAW264.7 cells, while NF-κB p65 was mainly distributed in the cytoplasm after AE administration (Figure 5C), indicating that AE represses the nuclear translocation of p65. In conclusion, AE inhibits the NF-κB signal pathway with better inhibition than DEX (*p* > 0.05).

### AE alleviates the inflammation response in LPS-yolk microinjected zebrafish

**FIGURE 6:**
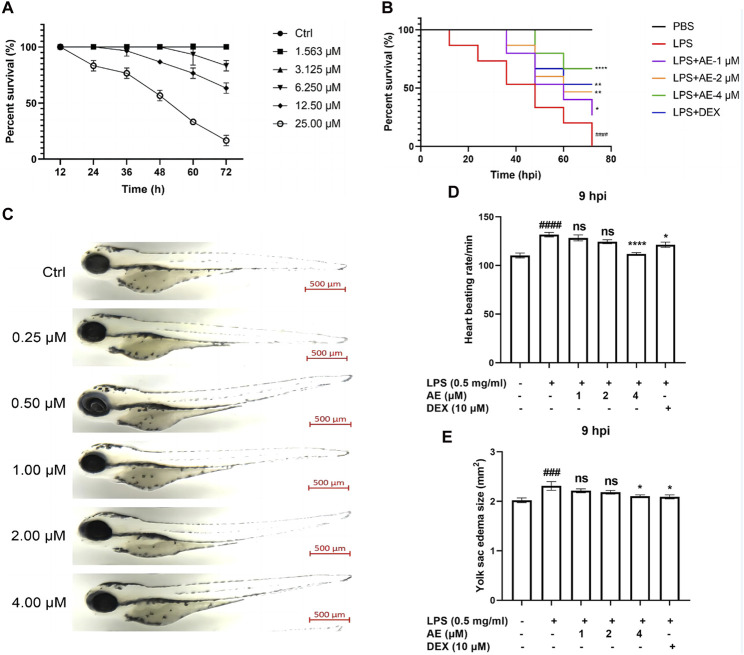
The protective effect of AE on zebrafish larvae microinjected with LPS. **(A)** 3 dpf larvae (n = 20) were immersed with different concentrations of AE for 72 h. The survival rate of zebrafish larvae was observed under a microscope **(B)** Lateral view of 3 dpf zebrafish larvae treated with AE for 24 h. Scale bar, 500 μm. **(C)** 3 dpf larvae (n = 15) were yolk-microinjected with PBS or LPS, and then treated with AE or DEX. Mortality was monitored until 72 hpi. **(D)** The heart-beating rate was counted at 9 hpi. **(E)** After 9 hpi the edema area of the yolk sac was tracked. Data were represented as mean ± SEM (n = 3), ^*^ *p* < 0.05; ^**^ *p* < 0.01; ^***^ *p* < 0.001;^****^ *p* < 0.0001; ns, no significance, compared with each’group. ^##^ *p* < 0.01; ^###^ *p* < 0.001; ^####^ *p* < 0.0001; compared with Ctrl group.

To evaluate the potential toxicity of AE in zebrafish, the mortality and morphological changes of 3-dpf zebrafish larvae immersed with different concentrations of AE were recorded every 12 h. Zebrafish larvae died in 6.250–25.00 μM AE treatment in a concentration-dependent manner, and zebrafish larvae survived in 1.563 and 3.125 μM AE treatment (Figure 6A). In addition, zebrafish larvae did not exhibit spinal curvature, pericardium enlargement, or cardiac bleeding following treatment with 1, 2, and 4 μM AE for 24 h (Figure 6B). This suggests that the drug within this range was not noticeably toxic to zebrafish larvae, and therefore a concentration gradient of 1, 2, and 4 μM was chosen for further experimentation. Further, administration of AE increased the survival rate of zebrafish larvae as the survival rate was improved up to 67.7% after 72 h post-injection with 4 μM AE (Figure 6C). Additionally, the stimulation of LPS can enhance the heart rate which is a sign of inflammation in zebrafish larvae. The heart rate of juvenile zebrafish experienced a notable increase at 9 hpi. However, with the intervention of AE, the heart rate gradually returned to normal. Specifically, 4 μM AE (*p* < 0.0001, F(5, 85) = 13.47) and DEX (*p* < 0.05) were able to completely restore the heart rate to the control level (Figure 6D). Furthermore, AE [*p* < 0.05, F (5, 71) = 4.205] and DEX (*p* < 0.05) were found to effectively reduce yolk sac edema caused by LPS (Figure 6E). In sum, AE exhibits potential in mitigating endotoxin-induced damage in zebrafish.

### AE inhibits the recruitment of inflammatory cells in LPS-yolk microinjected zebrafish

**FIGURE 7:**
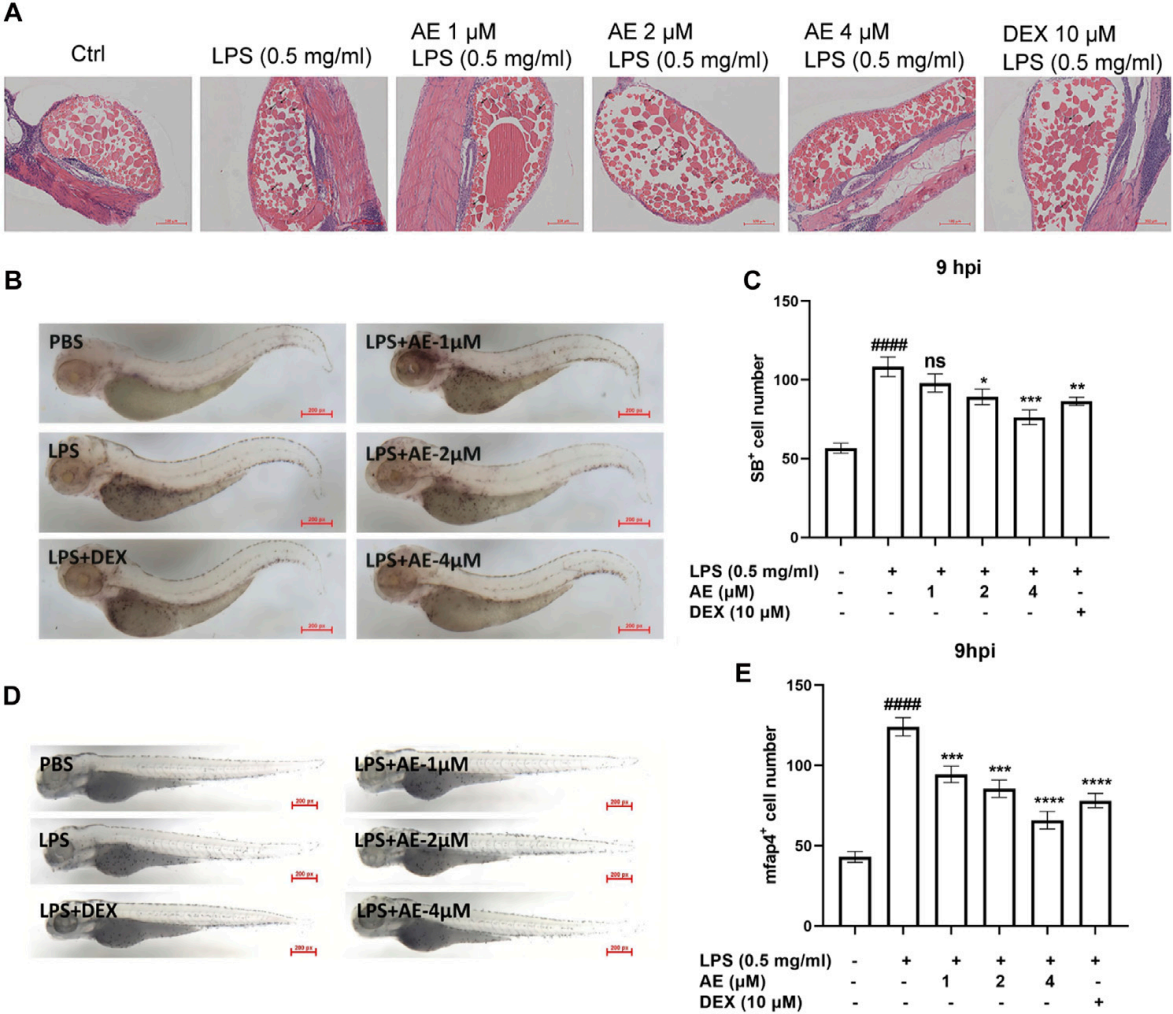
AE inhibits inflammatory cells recruitment in LPS-yolk microinjected zebrafish. **(A)** 3 dpf larvae (*n* = 15) were yolk-microinjected with PBS or LPS, and then treated with AE or DEX. The larvae were stained by H&E and taken pictures with a microscope, 20×. The black arrows represent inflammatory cells. **(B)** SB staining was performed at 9 hpi, 25×. **(C)** Statistical analysis of neutrophils in yolk sac of zebrafish larvae. **(D)** Whole-mount in situ hybridizations of mfap4 in zebrafish embryos at 9 hpi, 25×. **(E)** Statistical analysis of macrophages in yolk sac of zebrafish larvae. Data were represented as mean ± SD (*n* = 3), *p < 0.05; **p < 0.01; ***p < 0.001; ****p < 0.0001; ns, no significance, compared with each’ group. ##*p* < 0.01; ###*p* < 0.001; ####*p* < 0.0001; compared with Ctrl group.

The H&E staining assay showed that in the zebrafish larvae stimulated by LPS, many inflammatory cells infiltrated into the yolk sac, and AE treatment could significantly improve this situation (Figure 7A). Additionally, SB staining assay and *in situ* hybridization were performed to observe the recruitment of neutrophils and macrophages in zebrafish. LPS microinjection induced neutrophils recruitment to the yolk sac site [*p* < 0.0001, F (5, 76) = 14.62], whereas 4 µM AE (*p* < 0.001) and DEX (*p* < 0.01) immersion impaired the recruitment of neutrophils towards the zebrafish yolk sac site (Figures 7B, C). Following LPS stimulation, the positive signal of the mfap4 macrophage-specific marker significantly increased in comparison to the control group [*p* < 0.0001, F (5, 60) = 37.52]. On the other hand, the application of 4 µM AE (*p* < 0.0001) and DEX (*p* < 0.0001) led to a reduction in macrophage recruitment (Figures 7D, E). In sum, these results indicate that AE inhibits the recruitment of inflammatory cells.

### AE binds to and inhibits TLR4

**FIGURE 8:**
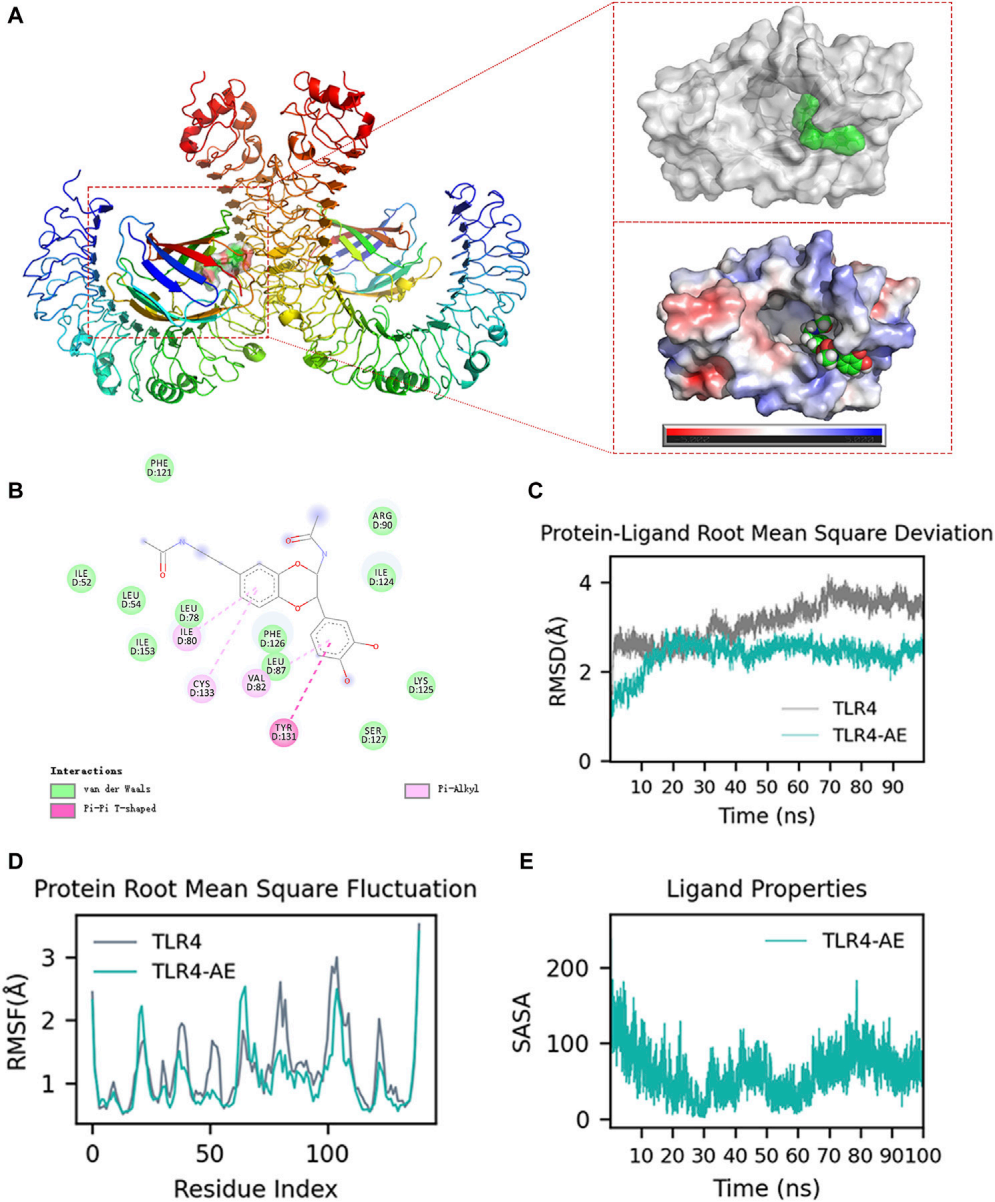
Molecular docking and molecular dynamics simulation analysis. **(A)** AE Docking Pockets and Electrostatics Analysis in Ribbon and Surface Forms. **(B)** Ligand-receptor interactions. **(C)** Root means square deviation of proteins and ligands over time. **(D)** Root means square fluctuation of protein residue index. **(E)** solvent-accessible surface area curve with time.

We aimed to explore whether AE could inhibit the key targets in the upstream TLR4-Myd88-TRAF6-TAK1 complex by computational simulation methods. We found that AE could bind to TLR4 with the lowest binding energy (−59.68 kcal/mol) (Supplementary Table S2). The ribbon and surface representations in Figure 8A illustrate the binding between TLR4 and AE in three dimensions. AE preferentially bound to the intermediate cavity region of the TLR4 D chain with negative charge, demonstrating that AE conformed to the shape and electrostatic potential of the negative-charge region and thus occupied the lowest energy state. Additionally, the particular interactions between the active center residues of TLR4 and AE, such as hydrogen bonds, π-π stacking, π-alkyl and van der Waals interactions, is showed in Figure 8B. As the D-chain cavity region is also identified as the primary structural domain of LPS binding, we suggest that AE impacts TLR4-Myd88-TRAF6-TAK1 signaling by completely interacting with TLR4 Chain D. We further confirmed the AE-TLR4 interaction using molecular dynamics simulation. The conformational stability of the TLR4 and AE along with the molecular dynamics trajectories was evaluated from the root mean square deviation (RMSD) plots. Throughout the simulation, AE was able to maintain a comparatively stable conformation for the full 100 ns in the simulations with TLR4 and AE (Figure 8C). Furthermore, the root means square fluctuation (RMSF) values of the AE-TLR4 complex and TLR4 were similar during the 100 ns simulation time, suggesting that AE binding does not cause significant perturbation to the amino acid residues of TLR4 Chain D (Figure 8D). In addition, we examined the interaction between AE and the key residues of TLR4 throughout the simulation process by performing contact analysis (Supplementary Figures S2A, B). Notably, TLR4 Ser127 mainly maintained the connection with AE’s OH through H-bond residue for a long time (Supplementary Figure S2A), indicating that SER127 is a critical amino acid residue that affects the stability of TLR4-AE binding. Finally, solvent accessible surface area (SASA) is used to measure the degree of exposure of molecules in solution, and to evaluate how their conformational changes affect their surface exposure. Figure 8E shows the change of SASA values after AE binds to TLR4. As simulation time increases, the SASA displayed periodic fluctuations. It reached the lowest value at 30 ns, before slowly rising and reaching a trough at 60 ns and then rising again and reaching a valley. Despite some fluctuations, the SASA remained within a relatively low fluctuation range. This indicates that molecules underwent slight conformational changes, which may be linked to the continuous dissociation and binding of AE with TLR4.

## Discussion

Natural products are an important source for exploring the potential of anti-inflammatory activity. The Artemisia genus, renowned for its chemical and biological diversity, has garnered significant attention due to the discovery and isolation of the promising antimalarial compound artemisinin (Sailike et al., 2022). *A. vulgaris* L., a key medicinal herb within this genus, boasts a rich array of volatile oils, sesquiterpene lactones, and other active constituents (Olennikov et al., 2018). These compounds confer Artemisia vulgaris with notable anti-inflammatory properties by inhibiting the synthesis of inflammatory mediators, scavenging free radicals, and modulating immune responses (Jiang et al., 2019). Consequently, Artemisia vulgaris holds considerable potential and advantages in the exploration of novel anti-inflammatory drugs. For instance, *A. vulgaris* L. and its sesquiterpene compound, dehydromatricarin A, can significantly reduce the inflammatory cell infiltration and the expression of TNF-α and IL-6 in bronchoalveolar lavage fluid (Shin et al., 2017). Several sesquiterpenoids isolated from *A. vulgaris* L. showed inhibitory activity against NO and COX-2 (Xue et al., 2018; Zhang and Lv, 2019). Isoeupatoxin and Jaceosidin can inhibit COX-2 expression and significantly reduce TNF-α, IL-1β and PGE2 levels (Min et al., 2009). The sesquiterpene lactone AE extracted from *A. vulgaris* L. has been classified as guaiacane and contains 15 carbon atoms and three isoprene units. In addition, various spectroscopy techniques such as NMR, HRESIMS, IR, X-ray and UV were employed to identify the AE (Liu et al., 2022). The purity of AE was determined to be over 98% through three different elution systems using HPLC. In this study, we found that AE could significantly inhibit the production of inflammatory mediators (such as NO and PGs) and cytokines (such as TNF-α and IL-6) in LPS-induced RAW264.7 cells, and it could also reduce the formation of oxidative damage substances (such as ROS) compared to DEX, providing preliminary anti-inflammatory evidence for AE. The selection of DEX as a positive control to evaluate the anti-inflammatory effects of AE is predicated on its mechanism of action, which involves the inhibition of the synthesis and release of pro-inflammatory mediators such as prostaglandins, leukotrienes, and cytokines (including TNF-α, IL-1, and IL-6). DEX is known for its potent and broad-spectrum anti-inflammatory properties, making it widely used in clinical and experimental research (Soltani et al., 2023). It is also considered a standard for assessing the efficacy of new anti-inflammatory drugs (Madamsetty et al., 2022).

Next, we sought to demonstrate the anti-inflammatory efficacy of AE in zebrafish. LPS-induced zebrafish inflammation is usually established by non-invasive immersion of zebrafish embryos in the egg water containing LPS or by microinjection into the yolk sac, which induces an increase in the number of zebrafish immune cells and leads to an increase in the expression of inflammatory factors such as ROS, TNF-α and IL-6 (Yang et al., 2014; Ko et al., 2017). Excessive recruitment of neutrophils can also aggravate tissue damage and may lead to a variety of diseases (White et al., 2014). Therefore, therapeutic drugs that reduce neutrophil accumulation may help to ameliorate inflammation. In the present study, AE reduced the mortality and leukocyte infiltration of zebrafish stimulated by LPS. Post-treatment with AE, there is a marked reduction in the recruitment of neutrophils and macrophages, with the effects being significantly more pronounced than those of DEX. This demonstrates AE’s robust anti-inflammatory activity in zebrafish. This finding suggests that AE, as a secondary metabolite of *A. vulgaris* L., exhibits significant anti-inflammatory activity within the body. Furthermore, it implies that *A. vulgaris* may serve as a promising source for discovering new bioactive compounds. This aligns with previous reports, Chen et al. isolated a significant number of sesquiterpene lactones from *A. vulgaris* L. and discovered that some of these compounds exhibit greater NO inhibition compared to dexamethasone (Chen et al., 2022). Jitendra Pandey found that extracts from three different altitudes of *A. annua* leaves possess anti-inflammatory properties (Pandey et al., 2021). Bernatoniene J have discovered that extracts from *A. vulgaris* contain a higher concentration of phenolic compounds compared to those from *A. annua.* Additionally, the extracts from *A. vulgaris* exhibit more pronounced activity against glioblastoma stem cells (Bernatoniene et al., 2024). Another study has demonstrated that extracts from *A. annua*, *A. vulgaris*, and *Artemisia pontica* exhibit analgesic and anti-inflammatory effects in animal models. However, *A. vulgaris* maintains a distinct advantage (Ivanescu et al., 2021).

NF-κB is a classical regulator of inflammation occurrence and development. Sesquiterpene lactones have been shown to modulate various aspects of NF-κB signal pathway activation. For instance, parthenolide inhibits the activation of NF-κB by targeting cytoplasmic signal transduction factors (Li et al., 2018). Moreover, costunolide effectively prevented the degradation of IκBα/β in MDA-MB-231 cells (Choi et al., 2005). Helenalin attenuated the DNA binding activity of NF-κB/p65 by alkylating cysteine residues in the p65 DNA binding domain (Chen et al., 2017; Xiong et al., 2022). In addition, our data demonstrated that AE not only inhibits the phosphorylation of key proteins, such as IKK, IκBα and NF-κB p65, in the NF-κB signal pathway, but also inhibits the nuclear translocation of NF-κB p65.

The TLR4-Myd88-TRAF6-TAK1 signaling is a molecular pathway that mediates the inflammatory response to LPS (He et al., 2016). TLR4 recognizes LPS and activates the adaptor protein Myd88, which then recruits the E3 ubiquitin ligase TRAF6 and activates the kinase TAK1, resulting in the activation of downstream kinases IKK and MAPK to adjust the subsequent transcription factors NF-κB (Georg et al., 2020; Liu et al., 2020; Su et al., 2021; Lu et al., 2022). We employed molecular docking and molecular dynamics simulations to further scrutinize the mode of action. The combined MM/GBSA and contacts data indicate that AE exhibits a stable complex structure and strong affinity for TLR4 Chain D, implying that AE’s anti-inflammatory property is likely due to its attachment with TLR4 Chain D. Furthermore, Arg90 and Ser127 are the principal amino acid sites responsible for the AE-TLR4 interaction. Considering that Arg90 and Ser127 are also critical for the binding of LPS to TLR4, these results strongly suggest that AE impedes the activation of TLR4 by competing binding with LPS, which subsequently blocks TLR4-Myd88-TRAF6-TAK1 signal transduction. However, further *in vitro* experiments are required to confirm these findings. Taken together, our study demonstrated that the new sesquiterpene lactone AE possesses notable anti-inflammatory properties both *in vitro* (RAW264.7 cells) and *in vivo* (zebrafish larvae). We further elucidated that AE may exert anti-inflammatory effects by inhibiting the activation of TLR4-MAPK/JAK/STAT-NF-κB pathways. A limitation of this study is the small sample size (n = 3 per group), which may affect the generalizability of the findings. However, future works are encouraged to evaluate whether AE can aid the fight against LPS-stimulated inflammatory diseases in higher organisms such as mice and rats with larger sample sizes.

## Conclusion

This study highlights the therapeutic potential of AE, a natural sesquiterpene, by elucidating its efficacy as a potent anti-inflammatory agent. Additionally, the present study investigated the effect of AE on inflammatory pathways. And AE enriches the repertoire of bioactive anti-inflammatory constituents in *A. vulgaris* L.

## Data Availability

The raw data supporting the conclusions of this article will be made available by the authors, without undue reservation.

## Abbreviations

AE, Artemvulactone E; CC50, 50% cytotoxic concentration; COX-2, Cyclooxygenase-2; iNOS, inducible nitric oxide synthase; JAK, Janus kinase; LPS, lipopolysaccharide; MAPK, mitogen-activated protein kinase; NF-κB, Nuclear factor-κB; Hpf, hours post-fertilization; Dpf, days post-fertilization; RMSD, root mean square deviation; RMSF, root mean square fluctuation; ROS, reactive oxygen species; SASA, solvent accessible surface area; STAT, signal transducer and activator of transcription; TLR4, Toll-like receptor 4.
