# Supplementary material for: Artemvulactone E isolated from Artemisia vulgaris L. ameliorates lipopolysaccharide-induced inflammation in both RAW264.7 and zebrafish model
Source: Front Pharmacol. 2024 Jul 18;15:1415352. doi: 10.3389/fphar.2024.1415352 (PMC11291208; doi:10.3389/fphar.2024.1415352)
Supplement: Supplementary file 1 [file DataSheet1.DOCX]

**Supplemental information**

**Artemvulactone E isolated from *Artemisia vulgaris L.* ameliorates lipopolysaccharide-induced inflammation in both RAW264.7 and zebrafish model**

**Supplemental Figures**


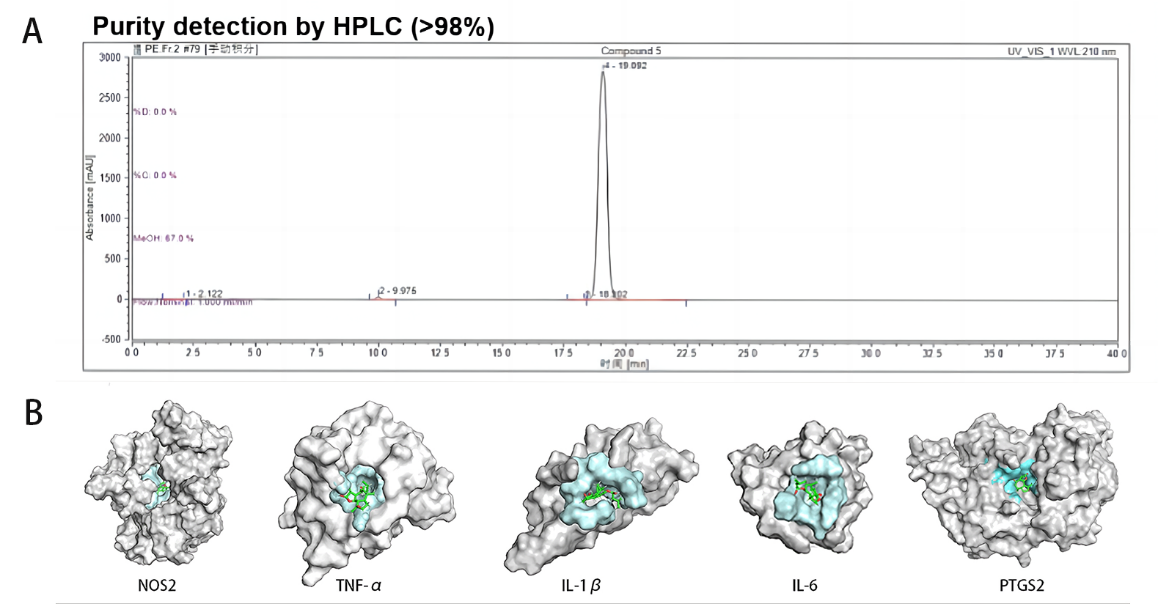


**Figure S1.** HPLC chromatogram of AE.


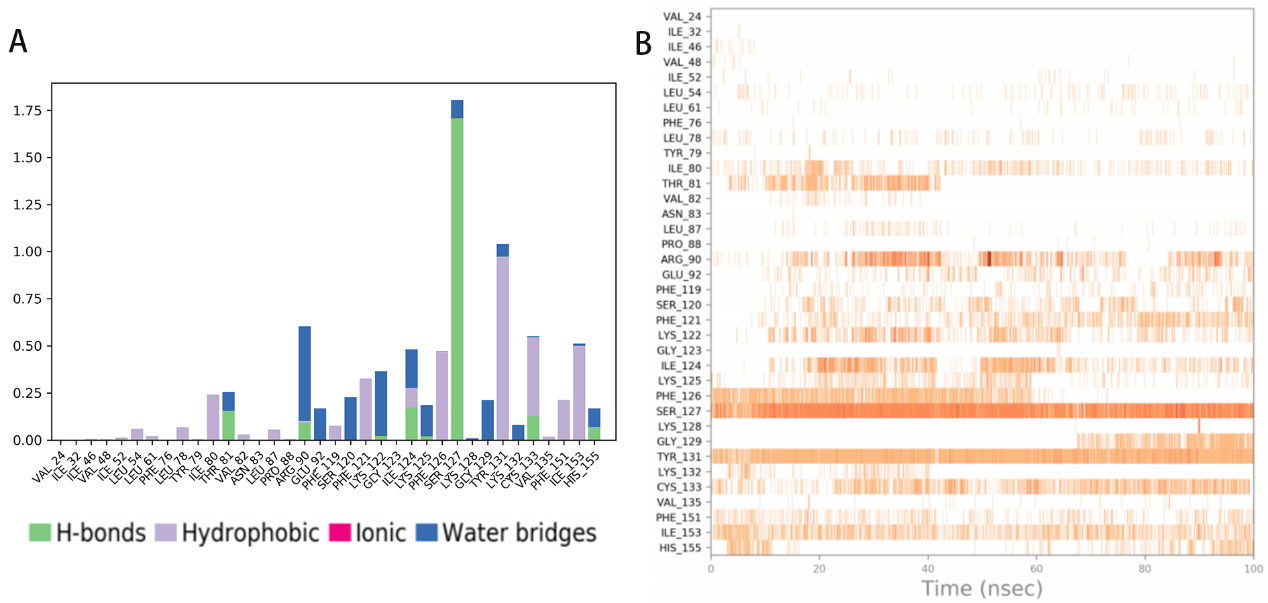


**Figure S2.** Protein-ligand contact analysis. (A) types of contact interactions. (B) Contact of ligand with residues during over time.

**Table. S1** Primer sequences for RT-qPCR analysis

| Genes | Sense (5′-3′) | Anti-sense (5′-3′) |
| --- | --- | --- |
| *GAPDH* | GTCATTGAGAGCAATGCCAG | GTGTTCCTACCCCCAATGTG |
| *TNF-α* | GGGAGCAAAGGTTCAGTGAT | CCTGGCCTCTCTACCTTGTT |
| *IL-1β* | GAGCCTGTGTTTCCTCCTTG | CAAGTGCAAGGCTATGACCA |
| *IL-6* | CTGACAATATGAATGTTGGG | TCCAAGAAACCATCTGGCTAGG |
| *NOS2* | AAGCAGCTGGCCAATGAG | CCCCATAGGAAAAGACTGCA |
| *PTGS-2* | ATTCCAAACCAGCAGACTCATA | CTTGAG TTTGAAGTGGTAACCG |

**Table. S2** Docking scores and free binding energy (MM/GBSA)

|  | TLR4 | MYD88 | TRAF6 | TAK1 |
| --- | --- | --- | --- | --- |
| Docking scores | -9.629 | -6.223 | -4.549 | -8.816 |
| MMGBSA(kcal/mol) | -59.68 | -47.36 | -33.78 | -46.50 |
